# Non-Uniform Deployment of LWSN for Automated Railway Track Fastener Maintenance Robot and GA-LEACH Optimization

**DOI:** 10.3390/s25185611

**Published:** 2025-09-09

**Authors:** Yanni Shen, Jianjun Meng

**Affiliations:** 1 School of Mechanical Engineering, Lanzhou Jiaotong University, Lanzhou 730070, China; 13220039@stu.lzjtu.edu.cn; 2 Institute of Mechanical and Electrical Technology, Lanzhou Jiaotong University, Lanzhou 730070, China; 3 Gansu Engineering Technology Research Center of Logistics and Transportation Equipment Informatization, Lanzhou 730070, China; 4 Gansu Logistics and Transportation Equipment Industry Technology Center, Lanzhou 730070, China

**Keywords:** wireless sensor network, linear, routing protocol, energy consumption balance, non-uniform node deployment, QoS

## Abstract

WSNs are an important component of the Internet of Things (IoT), and the research on their routing protocols has always been a hot topic in academia. However, in ARTFMRs’ collaborative operation along railway lines, there are common problems such as energy holes, high latency, and uneven energy consumption in LWSNs. To address these issues, this paper proposes a genetic algorithm-optimized energy-aware routing protocol (GAECRPQ). Firstly, a non-uniform deployment strategy of three-line isosceles triangles is constructed to enhance coverage and balance node distribution. Secondly, an energy–distance adaptive weighting mechanism based on a genetic algorithm is introduced for cluster head (CH) selection to reduce energy consumption in hotspots and extend the network lifetime. Finally, a task-aware TDMA dynamic time slot allocation method is proposed, which incorporates the real-time task status of ARTFMRs into communication scheduling to achieve priority transmission under latency constraints. The simulation results show, that compared with six unequal clustering protocols—EADUC, EAUCA, EBUC, EEUC, LEACH, and LEACH-C—the three-line isosceles triangle deployment has a wider coverage area, and the GAECRPQ protocol increases the network lifetime by 7.4%, the lifetime by 40%, and reduces the average latency by 55.77%, 53.07%, 47.61%, 39.87%, 52.08%, and 50.48%, respectively. This verifies that GAECRPQ has good performance in terms of network lifetime and energy utilization efficiency, providing a practical solution for the collaborative operation of ARTFMRs in railway maintenance scenarios.

## 1. Introduction

With the accelerated advancement of the global railway transportation sector, ensuring the safety and maintenance efficiency of track infrastructure has become a pivotal concern for maintaining operational reliability and improving overall transportation performance. Railway track bolts, as essential connecting components within the rail system, are highly susceptible to loosening or damage, which may result in serious consequences such as track misalignment, structural deformation, or even catastrophic train accidents [1,2]. Therefore, the real-time monitoring and timely maintenance of bolt conditions carry substantial engineering significance and socioeconomic value. Traditional manual inspection approaches are increasingly constrained by high labor costs, low inspection efficiency, and limited adaptability to harsh and dynamic railway environments. Consequently, they can no longer fulfill the growing demands for high-frequency scheduling and stringent safety assurance in modern railway operations. In response to these challenges, the development and deployment of Automated Railway Track Fastener Maintenance Robots (ARTFMRs) have provided promising solutions, offering enhanced operational efficiency, precision, and intelligence for track maintenance tasks. However, a key technical challenge remains: ensuring that ARTFMRs can maintain stable and efficient performance under the complex and dynamic conditions of real-world railway environments. In particular, enabling these systems to perceive and transmit real-time status information of track bolts remains a pressing issue within the broader context of intelligent railway operation and maintenance. Addressing this challenge is essential for the realization of autonomous, safe, and resilient railway infrastructure systems.

Wireless Sensor Networks (WSNs), as a pivotal enabling technology, have attracted widespread international interest in recent years and have exerted a substantial impact on industrial applications and daily life. As a critical component of the Internet of Things (IoT) infrastructure, WSNs employ a distributed and self-organizing architecture capable of high-precision, reliable, and real-time data acquisition and transmission in dynamic and complex environments. Providing a robust foundation for a wide range of intelligent applications, WSNs substantially enhance system-level perception, situational awareness, and intelligent decision-making. Their broad deployment in areas such as environmental monitoring, industrial automation, intelligent transportation, and infrastructure maintenance underscores their essential role in modern technological ecosystems [3]. In recent years, propelled by ongoing advancements in wireless communication technologies—especially the convergence of sixth-generation (6G) networks and the IoT—the deployment of WSNs has progressively extended into an increasingly diverse array of application domains [4,5,6], including smart agriculture, healthcare, smart homes, environmental monitoring, intelligent buildings, and military reconnaissance [7,8,9,10,11,12,13,14,15,16,17,18,19]. These diverse applications not only facilitate the intelligent transformation of various industries but also establish a robust foundation for the development of a fully interconnected intelligent society.

In summary, the accelerated advancement and extensive deployment of WSNs are anticipated to become progressively more integral to the architecture of next-generation intelligent systems. With ongoing enhancements in the capabilities and efficiency of WSNs, they are poised to deliver increasingly intelligent, reliable, and high-precision services across diverse industrial sectors. In the context of railway maintenance, WSNs can facilitate real-time acquisition of critical data—such as bolt fastening status, ambient temperature and humidity, and vibration signals—through sensor nodes deployed along railway lines. This data can be wirelessly transmitted to ARTFMRs or backend control centers, thereby enabling remote monitoring, intelligent analysis, and collaborative decision-making. Consequently, the integration of WSNs provides robust technological support for the real-time monitoring of rail fastener conditions and the synchronized operation of ARTFMR systems.

However, the unique characteristics of railway environments pose significant challenges to WSNs in terms of node deployment and routing protocol design. Railway tracks typically exhibit a linear topology, with lengths far exceeding their widths, making traditional uniform deployment strategies ineffective in achieving adequate communication coverage and efficient data transmission. Due to these linear constraints, sensor nodes in railway WSNs are generally arranged in a line along the track, and most nodes are battery-powered. As energy replenishment is difficult in such scenarios, WSNs are prone to the “energy hole” problem [20]. Specifically, in multi-hop communication scenarios where data is transmitted toward the sink node, sensor nodes situated in proximity to the sink typically shoulder a disproportionately high forwarding load. This leads to accelerated energy depletion, premature node failures, and link disconnections, ultimately compromising the connectivity and stability of the entire network. Therefore, designing a rational node deployment model that mitigates the energy hole phenomenon has become a critical issue in railway WSN research. In addition, uneven energy consumption among nodes during network operation can lead to localized power exhaustion, significantly shortening the overall network lifetime. Thus, achieving balanced energy consumption across different regions, while maintaining sufficient coverage and minimizing deployment and maintenance costs, has become a key objective in the development of node deployment strategies. Furthermore, the railway environment itself presents additional challenges: complex terrain variations, electromagnetic interference from high-speed trains, and limited energy availability impose stringent requirements on data transmission. WSNs deployed in such settings must meet simultaneous demands for low latency, high reliability, and energy efficiency. In this context, the strategic deployment of sensor nodes and the development of energy-efficient, high-performance routing protocols have become critical research challenges in overcoming practical application barriers of WSNs within ARTFMR systems operating in railway environments.

From the dual perspectives of mitigating the “energy hole” phenomenon and minimizing transmission delay, this paper designs a three-line Linear Wireless Sensor Network (LWSN) topology tailored to the characteristics of railway environments, specifically for the ARTFMR system. Furthermore, an energy-efficient clustering routing protocol is proposed, based on a comprehensive evaluation of node quality metrics. The primary contributions of this study are summarized as follows:

(1) In response to the deployment requirements of LWSNs for the ARTFMR system in railway scenarios, this paper proposes the Three-Line Isosceles Triangle Linear Deployment Strategy (TLITLDS). Through the placement of sensor nodes on both sides of the track as well as along the central axis, this deployment strategy substantially improves data collection accuracy and communication reliability, while concurrently minimizing coverage gaps in node distribution.

(2) An efficient and robust routing protocol is proposed to improve data transmission performance in LWSNs deployed within railway scenarios. The proposed protocol enhances transmission reliability and mitigates network congestion, while substantially extending the lifetime of sensor nodes, thereby ensuring the long-term reliability and stability of the system. The integration of the TLITLDS with the proposed efficient routing protocol effectively resolves the challenges related to communication reliability and energy efficiency in railway environments, thereby significantly enhancing the comprehensive performance of the ARTFMR system.

(3) To tackle the issue of accelerated energy exhaustion in certain cluster heads (CHs)—which can compromise network stability and shorten the system lifetime—a dynamic cluster head (CH) rotation mechanism is introduced. This strategy integrates both the remaining energy of nodes and their proximity to the sink node as critical factors in guiding the selection of CHs. In each election round, an adaptive weighting scheme is employed to fuse the average residual energy and proximity of each node to the sink node, thereby achieving an optimized balance between energy sustainability and transmission performance. A genetic algorithm (GA) is then leveraged to optimize the CH selection probabilities, facilitating dynamic role reassignment and spatially balanced CH distribution. This approach significantly mitigates uneven energy consumption, improves the uniformity of CH placement, and reduces the likelihood of network performance degradation caused by early failures in specific regions.

(4) In view of the fact that the traditional TDMA scheduling strategy usually adopts a fixed time slot allocation method in the data transmission process, without fully considering the actual load and energy state of nodes, resulting in low resource utilization, a method combining the priority of ARTFMR task status is proposed to optimize the TDMA scheduling priority, achieving a task-driven communication scheduling strategy. This strategy not only meets the delay constraints but also improves the real-time response capability and energy utilization efficiency of the system.

The remainder of this paper is structured as follows: Section 2 reviews the related work pertinent to this study. Section 3 introduces the proposed network model and energy consumption model. In Section 4, the design and implementation of the GA-Optimized Energy-Efficient Clustering Routing Protocol with QoS (Quality of Service) Constraints (GAECRPQ) are detailed, along with an analysis of its energy efficiency and algorithmic advantages. Section 5 presents a performance evaluation of the GAECRPQ protocol through simulation, comparing it against six benchmark protocols. Finally, Section 6 presents the concluding observations and outlines prospective directions for future research endeavors.

## 2. Related Work

To address the limitations of node deployment strategies in LWSNs, extensive research efforts have been undertaken by numerous scholars, leading to substantial advancements in the field.

Lalatendu et al. [21] proposed a novel WSN deployment scheme in response to the environmental monitoring requirements of longwall coal mines. This scheme studied and selected the node deployment mode with the minimum coverage density according to different coverage densities, which was used for sensor placement in the doorways of coal mines. By introducing a virtual force mechanism, it maximally reduced redundant overlapping areas while ensuring network connectivity, thereby effectively alleviating the “energy hole” problem in the LWSN. Simulation results demonstrate that the proposed method surpasses existing schemes in terms of regional coverage efficiency. Coert et al. [22] deployed a WSN to monitor mine safety, and by designing sensor devices and communication mechanisms, they constructed a WSN system suitable for underground mine environments. Experimental verification demonstrated that this system has excellent self-adaptive networking capabilities and communication reliability. Florian et al. [23] proposed a continuous coverage mechanism that reduces the overall energy consumption of WSNs by enhancing communication among sensors and optimizing the number and deployment of nodes, thereby improving the overall energy efficiency of the network. Rashmi et al. [24] proposed an enhanced k-means clustering approach aimed at achieving an optimal balance between network lifetime extension and load distribution in WSNs. The method partitions a large-scale monitoring area into multiple sub-clusters and employs the MCDS-MI and BG algorithms for CH selection. This strategy substantially enhances the network’s operational longevity and optimizes the efficiency of scheduling mechanisms. Haneen et al. [25] optimized the deployment density of WSNs to address the communication efficiency issue in pipeline (such as water, oil, and gas) monitoring. By deploying isolated sensors and reducing the node density, they effectively decreased the network latency and energy consumption and improved throughput performance under a linear topology. Simulation experiments verified its feasibility and effectiveness in energy-saving and low-cost leakage monitoring. Yang et al. [26] developed a linear topology-based WSN deployment strategy aimed at enhancing train operation safety and enabling real-time monitoring of train conditions within railway tunnels. To promote energy efficiency and prolong network longevity, the authors introduced a self-organizing network architecture and a routing protocol structured around a thick-line configuration. Simulation outcomes demonstrated that the proposed approach offers notable improvements in energy distribution uniformity and significantly extends the overall network lifespan.

Behera et al. [27] proposed an enhanced CH selection algorithm designed to prolong the operational lifetime of WSNs by effectively managing energy consumption within the network. Vikram et al. [28] proposed an improved algorithm integrating an independent recurrent neural network (IRNN) in response to the limitations of the LEACH routing protocol in terms of data redundancy and efficiency. This algorithm effectively enhances network performance and transmission efficiency and reduces data redundancy by enabling direct communication between CHs and base stations. Sureshkumar et al. [29] proposed a routing framework emphasizing node energy efficiency and trust evaluation to improve the overall energy performance of WSNs. Recognizing that secure data transmission via CHs can enhance both network lifetime and scalability—albeit at the cost of increased system complexity—the authors incorporated a CH selection mechanism based on the Adaptive Levy Walk Optimization (ALWO) algorithm. Simulation outcomes validated the efficacy of the proposed approach in achieving energy-aware and trustworthy routing. Prachi et al. [30] employed the Butterfly Optimization Algorithm (BOA) to enhance the selection of CH groups, aiming to minimize overall energy expenditure and extend the operational lifespan of WSNs. The proposed approach integrates multiple evaluation criteria, including residual energy, inter-node distance, and node degree centrality, and introduces an energy-aware routing protocol. Simulation results confirm that the proposed method outperforms existing algorithms across multiple performance metrics. Sandeep et al. [31] designed an intelligent transportation system (ITS) framework based on a WSN to enhance the efficiency of information management in military area road transportation. To reduce nodes’ energy consumption, they proposed an intelligent clustering method that integrates a GA and the bat algorithm (BA), and they selected CH nodes with energy-saving and safety characteristics. Simulation outcomes indicate that the proposed method exhibits enhanced performance in terms of network stability period, prolonged operational lifetime, and improved data transmission efficiency. Biswa et al. [32] introduced an enhanced CH selection strategy for heterogeneous WSNs using a genetic algorithm-based approach named NCOGA. This scheme integrates adaptive crossover with a binary tournament selection mechanism and formulates a comprehensive fitness function by incorporating multiple parameters, including residual node energy, distance to the sink, neighbor node density, load balancing index, and a communication mode decision module (CMD). Simulation results demonstrate that the proposed algorithm achieves superior performance compared to benchmark methods, particularly in terms of stability duration, overall network lifespan, and data throughput. Fjolla et al. [33] introduced a QoS-driven routing scheme aimed at mitigating node energy consumption and minimizing network latency in WSNs. To further enhance energy efficiency and ensure reliable data transmission, a Time-Division Multiple Access (TDMA) mechanism was incorporated into the protocol design. Wang et al. [34] proposed a novel adaptive data rate (ADR) algorithm to address the issue of QoS degradation caused by the use of LoRa as mobile terminal nodes in smart agriculture. The performance of LoRa WAN networks in agricultural scenarios under different ADR algorithms was evaluated through simulation and field tests, verifying the reliability and adaptability of the proposed algorithm. Yang et al. [3] introduced an advanced intelligent routing algorithm, termed WOAD3QN-RP, to mitigate the challenges of high communication latency and limited network lifespan in WSNs. The approach employs the Whale Optimization Algorithm (WOA) for optimal CH selection and integrates a Double Deep Q-Network (D3QN)-based deep reinforcement learning framework to train intelligent agents capable of adapting to dynamic network topologies. This facilitates an effective trade-off between multi-hop routing efficiency and energy consumption. Experimental evaluations reveal that the proposed algorithm substantially outperforms conventional schemes with respect to network longevity, energy utilization, and transmission delay. Alam et al. [35] addressed the routing and communication challenges arising from high mobility in collaborative unmanned aerial vehicle (UAV) networks by proposing a joint trajectory, resource allocation, and relay selection framework (JTFR). Built upon a multi-agent deep reinforcement learning paradigm, JTFR maximizes a composite utility function through simultaneous optimization of trajectory planning, resource allocation, and relay selection. Extensive simulations demonstrate that JTFR significantly outperforms state-of-the-art schemes in terms of end-to-end delay, transmission quality, and energy consumption. Sun et al. [36] proposed a Multi-Agent Actor–Critic Reinforcement Learning (MACRL) algorithm that accelerates convergence via action-space compression and introduces a multi-objective reward mechanism to balance competing performance metrics. Comprehensive simulations corroborate the effectiveness of the proposed approach. Xiao et al. [37] addressed the challenge of simultaneously accommodating dynamic network environments and stringent QoS requirements in multipath traffic engineering by proposing a multi-agent collaborative algorithm built upon a hybrid Knowledge-Defined Networking (KDN) architecture. The approach employs parallel agent replication, periodic policy synchronization, and a scalable communication mechanism, integrated with a PPO-optimized dual actor–critic training framework, to substantially enhance scalability, QoS awareness, and stability. Extensive simulation results corroborate the superior performance of the proposed scheme.

The WSN sensor nodes along the railway are usually manually deployed, and their geographical locations, initial energy, and other parameters are known and fixed before deployment [38]. Most existing schemes still adopt either uniform or stochastic node deployment and subsequently employ fixed-threshold routing protocols. These approaches, however, are inherently incapable of capturing the anisotropic propagation characteristics and spatially varying traffic loads that typify linear railway environments. Consequently, cluster heads become geographically clustered, leading to pronounced energy imbalance across the network. Moreover, the absence of task-aware TDMA scheduling mechanisms precludes the real-time prioritization of critical data streams and coordinated channel access, further degrading communication efficiency. This paper, from the perspective of avoiding the “energy hole” phenomenon and reducing transmission delay, designs an LWSN topology based on the Three-Line Isosceles Triangle Linear Deployment Strategy (TLITLDS) for the ARTFMR system in the railway scenario and, on this basis, proposes a GA-Optimized Energy-Efficient Clustering Routing Protocol on QoS (GAECRPQ).

## 3. Problem Description and System Model

### 3.1. Problem Description

The LWSN is a typical topology in Wireless Sensor Networks (WSNs), widely applied in monitoring scenarios such as tunnels, highways, riverbanks, and power transmission lines, which feature long-distance linear distribution [21,22,39]. In this model, sensor nodes are usually deployed along the linear area to continuously perceive the environmental conditions and event changes in the target area. Although the actual linear areas in applications may have some degree of curvature or bends, in terms of perception coverage and data transmission, their network behavior is essentially consistent with that of an ideal linear structure. Therefore, in the modeling and algorithm design of this paper, it is assumed that the WSN is in an ideal linear deployment form to simplify problem analysis and improve modeling accuracy.

**Figure 1:**
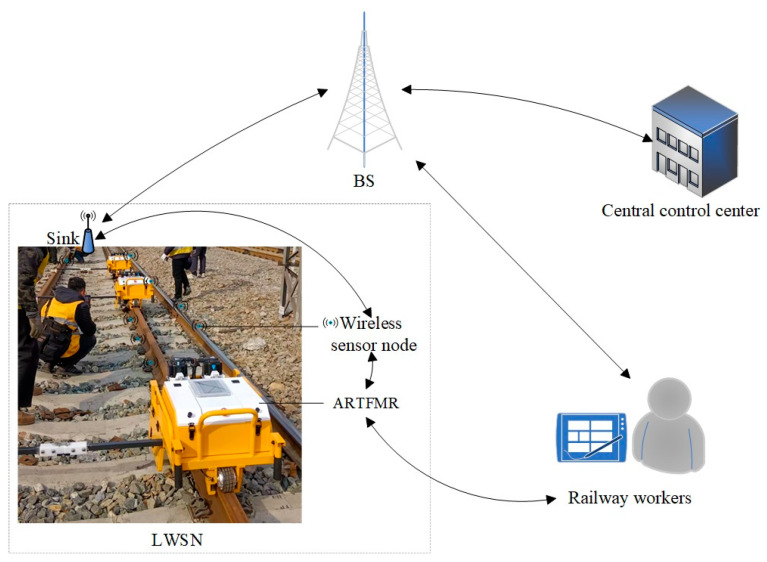
ARTFMR operation mode in railway scenarios.

In railway monitoring scenarios, the track structure typically presents as straight lines or large-radius arcs, with a standard lateral track width D of 1435 mm and a longitudinal length that can reach several kilometers, often spanning multiple geographical regions. This typical linear spatial topology characteristic poses higher requirements for the node deployment strategy, data transmission path planning, and energy management mechanism of LWSNs. Especially in the context of ARTFMRs participating in operation and maintenance tasks, to ensure the reliability of perception data and the real-time performance of communication, it is necessary to design a reasonable layout scheme for sensor nodes and corresponding routing protocol optimization mechanisms to effectively reduce the data transmission delay and network energy consumption of the system, and to extend the overall network life cycle. The mode of ARTFMR operation and perception collaboration in the railway scenario is shown in Figure 1. The daily operation and maintenance tasks are initially generated by the central control unit and transmitted to the BS via a designated communication link. Serving as the central information relay node, the BS subsequently disseminates task directives to both the sink nodes within the LWSN and the on-site operational staff. The sink nodes establish connections with the sensor nodes deployed along the track through multi-hop communication, forming a data perception and communication path, and achieving the distribution and synchronization of task information in the network. The ARTFMR receives task instructions through interaction with adjacent sensor nodes and achieves interconnection with the wireless network. At the same time, the operation personnel can also directly control and intervene in the ARTFMR through dedicated operation terminals, achieving human–machine collaborative operation and ensuring the precise execution and efficient response of operation and maintenance tasks.

### 3.2. Network Model

**Figure 2:**
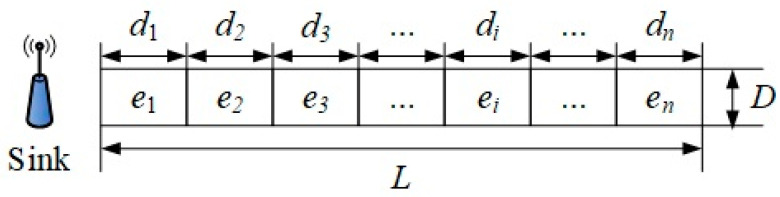
LWSN model of ARTFMR in railway scenario.

Assume that the LWSN is deployed within a rectangular region characterized by a longitudinal length *L* significantly greater than its width *D*, under the condition that L≥D. This area conforms to a typical linear spatial structure. *N* sensor nodes are deployed in the network, including a sink node located at one end of the rectangular area. All sensor nodes have the same initial energy reserve, a communication radius of *r*, and computing and communication capabilities at the beginning. It is also assumed that the sink node has unlimited energy and computing resources to perform central network scheduling and data aggregation tasks. During the operation of the network, sensor nodes are first allocated to different cluster structures according to the preset deployment strategy. Regular sensor nodes within each cluster are tasked with acquiring localized environmental data and transmitting the gathered information to their corresponding CH node. The CH node aggregates the incoming data and forwards it to the sink node via multi-hop communication, thereby enabling global data acquisition and consolidation of sensing information [40]. Based on the aforementioned assumptions and structural characteristics, the LWSN topology model is constructed as illustrated in Figure 2. The sink node is deployed on the left side of the entire LWSN, and the clusters are sequentially marked as $e_{1},e_{2},e_{3},\ldots ,e_{n}$, from near to far from the sink node.

For the LWSN relied on by the ARTFMR, its topological structure has typical spatial linear distribution characteristics. The spatial deployment of sensor nodes has a substantial impact on their energy consumption patterns, often resulting in uneven energy distribution across the network, which can subsequently trigger issues such as the formation of an “energy hole”. To more accurately analyze the communication and energy consumption characteristics of the LWSN in the ARTFMR’s collaborative operation in the railway scenario, the following assumptions are made regarding the track deployment environment in the modeling process of this paper:

(1) All sensor nodes within each cluster are homogeneous and deployed manually. Once deployed, their positions are fixed and they have no mobility. Their energy is supplied by an external power source, and they have strong local processing and computing capabilities.

(2) Node locations are specified within a two-dimensional Cartesian coordinate framework, and the inter-node distances are computed using the Euclidean metric. The energy consumption behavior of the nodes is modeled using the classical first-order radio energy dissipation model, which captures the energy dynamics during both data transmission and reception processes [41].

(3) The network comprises a total of *N* sensor nodes, each initialized with identical energy levels and assigned a unique identifier (ID) to facilitate efficient management and identification during routing formation and CH selection procedures.

(4) Only one sink node is deployed in the network, fixed on one side of the area. This node has an unlimited energy supply capacity and efficient data processing capabilities, and it does not participate in the CH rotation process.

(5) All nodes have data fusion capabilities and can adaptively adjust their transmission power based on channel conditions and the signal strength between nodes. Additionally, under certain conditions, ordinary nodes can assume the role of CHs, enabling a flexible dynamic rotation mechanism for CHs.

### 3.3. Energy Consumption Model

**Figure 3:**
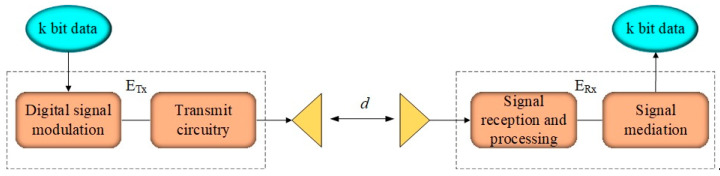
Energy consumption model of LWSN.

Since ARTFMRs typically operate in outdoor environments, their working scenarios are complex and changeable, with a large spatial span. The WSNs that they rely on face the practical challenges of long-term unattended operation and high maintenance difficulty. Once the energy of sensor nodes is depleted, timely replacement or recharging is typically unfeasible, thereby posing a significant threat to the stability and longevity of the network. In the operational phase of WSNs, energy consumption primarily arises from three functional modules: sensing, data processing, and wireless communication. Extensive research has demonstrated that, among these modules, the energy consumed during communication—particularly data transmission—constitutes the largest proportion of the total energy expenditure, significantly exceeding that of sensing and processing tasks [42,43]. Consequently, in practical energy consumption modeling and optimization design, the energy costs associated with sensing and data processing are typically neglected to streamline the analysis and concentrate on the dominant source of energy expenditure—wireless communication. Accordingly, this study adopts the two-ray ground-reflection model in WSNs [44] for constructing the energy consumption framework, as illustrated in Figure 3. Assuming that the data volume of a single transmission is *k* bits and the transmission distance is *d*, the energy consumption of a node to complete one round of data transmission is shown in Equation (1).

(1) $$E_{TX}(k,d)=kE_{elec}+k\varepsilon_{amp}d^{n}=\left\{\begin{array}{l} kE_{elec}+k\varepsilon_{fs}d^{\sigma }\;\;\;d<d_{0}\\ kE_{elec}+k\varepsilon_{mp}d^{\sigma }\;\;d\geq d_{0} \end{array}\right.$$

The energy consumption of a node receiving *k*-bit data is shown in Equation (2):

(2) $$E_{RX}(k,d)=kE_{elec}$$

In Equation (1), $E_{elec}$ represents the energy consumed by a node for sending or receiving 1 bit of data over a circuit; σ is the path loss factor, 2≤σ≤4; *d* is the actual data transmission distance; $d_{0}$ is the communication distance threshold, as shown in Equation (3); when the transmission distance is $d<d_{0}$, the energy consumption model is the free-space model; when the transmission distance is $d\geq d_{0}$, the energy consumption model is the multipath attenuation model.

(3) $$d_{0}=\sqrt{\frac{\varepsilon_{fs}}{\varepsilon_{mp}}}$$

where $\varepsilon_{fs}$ and $\varepsilon_{mp}$ are the energy consumption coefficients of the amplifier under different attenuation models.

## 4. Methodology

### 4.1. Non-Uniform Node Deployment with Energy Consumption Balance

Building upon the LWSN framework for ARTFMRs in railway environments illustrated in Figure 2, this study develops and analyzes a sensor node deployment strategy tailored to the linear characteristics of railway tracks. Firstly, sensor nodes need to be reasonably deployed in the railway environment monitoring area to achieve precise perception and real-time monitoring of key information such as the state of track bolts and environmental parameters [45,46,47]. As the foundation of WSN system construction, the rationality of node deployment will directly affect the coverage capability, communication quality, and energy consumption distribution of the network.

Suppose that the total length of the network is *L*, the total number of sensor nodes is *N*, and the entire network is divided into *n* cluster regions. For the *i*th cluster in this network, if it is the cluster region farthest from the sink node, then the energy consumption $E_{si}$ within the cluster mainly consists of three parts: (1) the energy consumption $E_{TX-fs}$ for the sensor nodes within the cluster to send data packets to the CH node, (2) the energy consumption $E_{RX-fs}$ for the CH node to receive the data sent by the sensor nodes, and (3) the energy consumption $E_{TX-fsi}$ for the CH node to forward the fused data to the next adjacent CH node after processing the received data. According to the energy consumption expressions for sending and receiving in the first-order wireless sensor network energy model (Equations (1) and (2)), the calculation formulae for the above three types of energy consumption are shown in Equations (4) to (6):

(4) $$E_{TX-fs}=kE_{elec}+k\varepsilon_{mp}d^{4}$$

(5) $$E_{RX-fs}=kE_{elec}$$

(6) $$E_{TX-fsi}=kE_{elec}+k\varepsilon_{mp}d_{i}^{4},i=1,2,3,\ldots ,n-1$$

In Equation (6), $d_{i}$ represents the distance from the *i*th CH node within the network to the (*i*−1)-th CH node.

Based on Equations (4) to (6), the intra-cluster energy consumption $E_{si}$ can be approximately obtained as shown in Equation (7):

(7) $$E_{si}=E_{RX-fs}+2E_{TX-fsi}=3kE_{elec}+2k\varepsilon_{mp}d_{i}^{4},i=1,2,3,\ldots ,n-1$$

When the data volume sent and received by sensor nodes within a cluster in the network is *k* bits each, and the communication distance between nodes is *d*, the total communication energy consumption $E_{n}$ corresponding to it is as shown in Equation (8):

(8) $$E_{n}=\sum_{i=1}^{n}E(k,d_{i})=(2n-1)kE_{elec}+k\varepsilon_{mp}\sum_{i=1}^{n}d_{i}^{4},\sum_{i=1}^{n}d_{i}\geq L$$

If the cluster is not the farthest cluster, in addition to the intra-cluster energy consumption $E_{si}$, the data sent by the previous CH also needs to be received and forwarded. Therefore, the intra-cluster energy consumption $E_{i}$ of the non-farthest cluster is as shown in Equation (9):

(9) $$E_{i}=3kE_{elec}+2k\varepsilon_{mp}d_{i}^{4}+(n-i)(2kE_{elec}+k\varepsilon_{mp}d_{i}^{4}),i=1,2,3,\ldots ,n-1$$

Theorem: Let the multivariate function $f(x_{i})=x_{1}^{m}+x_{2}^{m}+x_{3}^{m}+\ldots +x_{i}^{m}$, m≥2 (where $x_{i}$ is the independent variable of the multivariate function) have a minimum value under the constraint condition $\sum_{i=1}^{m}x_{i}\geq L$, if and only if it is $x_{1}=x_{2}=x_{3}=\ldots =x_{i}$. The extremum problem of the multivariate function with constraints is proven by the Lagrange multiplier method.

From the above theorem, it can be known that when $d_{1}=d_{2}=d_{3}=\ldots =d_{n}=L/n$ in Equation (9), the total energy consumption $E_{n}$ reaches its minimum value; that is, when the size of each sub-cluster is equal, the total energy consumption for inter-cluster data transmission is the smallest. Therefore, when the derivative result of Equation (8) is taken as 0, the optimal sub-cluster distance can be obtained as shown in Equation (10):

(10) $$d_{best}=\frac{L}{n}=\sqrt[{4}]{\frac{2E_{elec}}{3\varepsilon_{mp}}}$$

To achieve uniform average energy consumption across the network, the ratio of the total energy within each cluster to the number of constituent nodes should be maintained as equal as possible, as expressed in Equation (11):

(11) $$\frac{E_{i}}{N_{i}}=\frac{E_{n}}{N_{n}}$$

In Equation (10), $N_{i}$ represents the total number of nodes contained in the *i*th cluster, $N_{n}$ represents the total number of nodes in the network, and $E_{i}$ represents the total energy consumption of the nodes within the *i*th cluster.

The relationship between the number of nodes in the *i*th cluster and the farthest cluster is obtained from Equations (7), (9) and (11), as shown in Equation (12):

(12) $$N_{i}=\frac{E_{i}}{E_{n}}\cdot N_{n}=\left[1+\frac{(2n-2i)E_{elec}+(n-i)\varepsilon_{mp}d_{i}^{4}}{3E_{elec}+2\varepsilon_{mp}d_{i}^{4}}\right]\times N_{n}$$

The total number of the farthest intra-cluster nodes is obtained as shown in Equation (13), based on Equations (10)–(12):

(13) $$N_{n}=\left[\frac{13}{4n^{2}+9n}\right]N$$

The total number of nodes within the *i*th cluster is obtained as shown in Equation (14), based on Equations (10), (12) and (13):

(14) $$N_{i}=\left[\frac{13+8(n-i)}{4n^{2}+9n}\right]N$$

**Figure 4:**
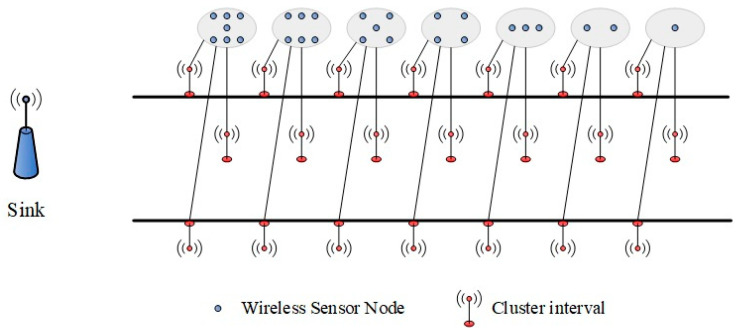
Node deployment model.

As depicted in Figure 2, regions situated closer to the convergence (sink) node contain a higher density of sensor nodes. In Figure 2, one block area represents one clustering interval; each clustering distance can be obtained according to the network scale by Formula (10). To ensure equitable energy distribution throughout the network, sensing nodes with varying deployment densities can be strategically allocated within each clustering region. The designed node deployment model is illustrated in Figure 4.

**Figure 5:**
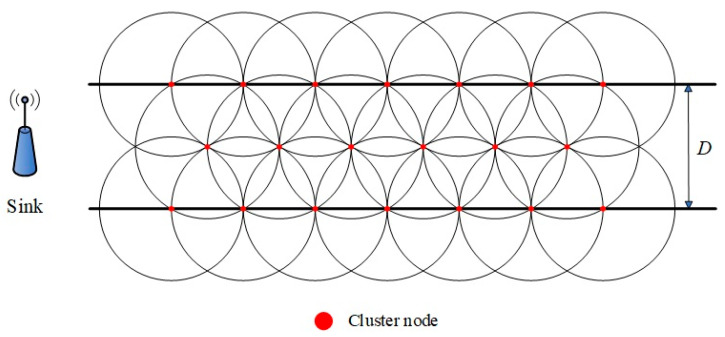
Node overlapping coverage deployment model.

In the typical linear topology structure along the railway, the deployment positions of sensor nodes are restricted by the actual track structure, and the nodes can only be set up at fixed sampling points on both sides and the centerline of the track. In response to this constraint, this paper designs a node overlapping coverage deployment model, as shown in Figure 5. This model deploys multiple sensor nodes in an overlapping manner at the sensing points in each cluster area, as needed to enhance node redundancy and network robustness. The total number of nodes in each sub-cluster is determined using Equation (14), and their deployment density is adjusted according to the corresponding load demands.

During the normal operation of the network, only one node at each sensing point is allowed to be in an active working state, while the rest of the overlapping deployed nodes remain dormant. Once the energy of the current working node is exhausted, the system automatically wakes up the next dormant node at that point to enter the working state. This cycle repeats itself until all of the overlapping nodes at that sensing point are “dead”. This rotation mechanism effectively extends the functional duration of the sensing point and reduces the risk of early energy depletion of a single node due to high-frequency operation.

This deployment strategy integrates overlapping node scheduling with hierarchical density control, specifically increasing the concentration of sensing nodes in clusters located near the sink node. This approach mitigates the “energy hole” issue arising from excessive data-forwarding burdens. Furthermore, it exhibits strong adaptability and scalability in enhancing network longevity and promoting balanced energy consumption across the entire system.

### 4.2. Improved Algorithm Description

An appropriately engineered routing protocol can significantly mitigate the “energy hole” issue within the network and extend its operational lifespan [48].

Leveraging the topological characteristics of LWSNs deployed along railway lines, this paper presents a Genetic Algorithm-Optimized Energy-Efficient Clustering Routing Protocol with Quality of Service Constraints (GAECRPQ). The proposed protocol is designed to holistically optimize the network’s energy consumption profile, improve data transmission efficiency, and substantially prolong the sensor network’s operational lifespan, thereby offering robust data sensing and communication support for the ARTFMR system.

During the operation of the GAECRPQ protocol, to ensure the node delay performance and avoid data collisions, it is considered to perform clustering through non-uniform node deployment when electing CHs.

Heinzelman et al. initially introduced the seminal LEACH (Low-Energy Adaptive Clustering Hierarchy) protocol in 2000 and subsequently validated its effectiveness through experimental implementation in 2002 [49]. As one of the earliest and most representative hierarchical routing protocols in the WSN field, LEACH has been widely applied in actual WSN systems and laid the theoretical foundation for the design of subsequent clustering routing protocols.

This protocol first introduced the idea of “randomized clustering” and proposed the concept of “round-based operation mechanism”, providing innovative ideas for the design of subsequent energy-saving protocols. LEACH was the earliest protocol to systematically introduce the clustering mechanism into the routing structure of WSNs. Its core objective is to achieve balanced energy consumption among nodes through periodic CH rotation and hierarchical data transmission, thereby effectively extending the network’s life cycle.

In the operational framework of the LEACH protocol, network activity is organized into discrete “rounds”, each comprising two primary phases:

(1) Cluster Formation Phase: CH nodes are selected through a probabilistic mechanism, and sensing nodes are organized to join clusters.

(2) Steady-State Phase: During this stage, cluster member nodes forward their sensed data to the designated CH, which aggregates the received information and subsequently transmits it to the sink node.

This iterative clustering and communication process substantially improves the network’s energy efficiency and forms the foundational architecture upon which many subsequent enhanced protocols are developed.

In the conventional LEACH protocol, each sensor node contends for the role of CH with an equal probability *p*. The threshold function utilized in the CH selection process, as defined in Equation (15), regulates the likelihood of a node being selected as a CH during each round. This mechanism ensures a rotational selection of CHs across multiple rounds, thereby promoting a more balanced energy consumption distribution throughout the network.

(15) $$T(n)=\left\{\begin{array}{l} \frac{p}{1-p\cdot (r\;\mathrm{mod}\frac{1}{p})}\;\;\;n\in G\\ 0\;\;\;\;\;\;\;\;\;\;\;\;\;\;\;\;\;\;\;\;\;\;\;\;\;\;\;\;\;\;\;\;\;n\notin G \end{array}\right.$$

where *G* denotes the set of nodes that have not served as CHs in the preceding rounds, while *r* represents the current round index.

According to Equations (10) and (14), the network has been spatially divided into several clusters of different sizes, with varying numbers of nodes within each cluster. Under this structure, the traditional LEACH protocol uses a fixed probability mechanism to select the CH, which fails to fully consider the spatial positions and energy states of the nodes. This may result in a non-uniform distribution of CHs, leading to energy imbalance across the network and early depletion of certain nodes.

To ensure balanced energy consumption across the entire network, it is essential to implement a dynamically optimized CH selection strategy. This paper introduces a weighted evaluation framework that incorporates both the residual energy and spatial location of sensor nodes during each CH selection round. Specifically, an adaptive weighting factor is employed to integrate the node’s average residual energy with its distance to the sink node, thereby assessing its suitability for assuming the CH role. Subsequently, a GA is utilized to refine and update the CH selection probabilities for each node, enabling dynamic role allocation and a more equitable CH distribution. Ultimately, nodes within each cluster determine their eligibility for CH candidacy using an enhanced threshold function. This strategy substantially enhances the fairness and energy-conscious efficiency of the CH selection process, thereby effectively prolonging the network lifespan and reinforcing the overall robustness of the system.

Factor A of the remaining energy of the node is calculated according to Equation (16). A higher value indicates that the node possesses greater residual energy.

(16) $$f_{1}=\frac{E_{res}(i)}{E_{ave}(i)}$$

where $E_{res}(i)$ represents the remaining energy value of node *i* in the current round, and $E_{ave}(i)$ represents the average remaining energy value of node *i* in the current round.

Factor $f_{2}$ of the node position is calculated according to Equation (17). A higher value signifies that the node is in closer proximity to the sink node, thereby increasing its likelihood of being selected as a CH.

(17) $$f_{2}=\frac{d_{\max}-d_{i}}{d_{\max}}\cdot \alpha_{i}$$

where $d_{i}$ represents the distance from node *i* to the sink, $d_{\max}$ represents the maximum distance in the network, and $\alpha_{i}$ represents the deployment area weight, with the middle line being 1.2, the side closer to the sink being 1.1, and the far side being 0.9.

The factors f affecting the selection of CHs are shown in Equation (18):

(18) $$f=\rho_{1}f_{1}+\rho_{2}f_{2}$$

where both $\rho_{1}$ and $\rho_{2}$ are weighting factors, used to adjust the relative importance of each influencing factor in the CH selection decision, thereby achieving a comprehensive balance between energy and distance factors. Here, $\rho_{1}+\rho_{2}=1$.

As the network undergoes successive operational cycles, disparities in energy consumption among individual nodes become progressively more pronounced. Therefore, it is imperative to adaptively adjust the weighting coefficients of the relevant factors to facilitate the selection of the most suitable CH. The formula for dynamically updating the weight coefficients is provided in Equation (19):

(19) $$\rho_{1}=E_{\max}(i)-(\frac{r}{R})\cdot [E_{\max}(i)-E_{\min}(i)]$$

where $E_{\max}(i)$ represents the maximum remaining energy of the surviving nodes in the cluster where node *i* is located in the current round, $E_{\min}(i)$ represents the minimum remaining energy of the surviving nodes in the cluster where node *i* is located in the current round, *r* represents the current round number, and *R* represents the total number of rounds.

The advantage of dynamically adjusting the weight coefficient according to Equation (19) lies in the fact that, as the network operation time increases, the remaining energy of the node has a greater impact on the selection of CHs, which can alleviate the problem of frequent selection and early death of CHs in hotspots. Accordingly, the CH selection mechanism utilized in the proposed GAECRPQ protocol can be formally represented by Equation (20):

(20) $$T(n)=\left\{\begin{array}{l} \frac{p(\rho_{1}f_{1}+\rho_{2}f_{2})}{1-p\cdot (r\;\mathrm{mod}\frac{1}{p})}\;\;\;n\in G\\ 0\;\;\;\;\;\;\;\;\;\;\;\;\;\;\;\;\;\;\;\;\;\;\;\;\;\;\;\;\;\;\;\;\;n\notin G \end{array}\right.$$

A genetic algorithm (GA) has the advantages of flexible coding methods, strong global search capabilities, and no requirements for the continuity and differentiability of the fitness function [50]. Therefore, an energy-aware adaptive CH selection mechanism based on a GA is developed in this study. The proposed mechanism aims to optimize the spatial distribution of CHs, improve energy efficiency across the network, and extend the operational lifetime of the LWSN in railway scenarios, all while maintaining robust network coverage performance.

Given a total of *N* deployed nodes in the network, each chromosome can be represented as a vector of length *N*, as shown in Equation (21):

(21) $$chromosome=[g_{1},g_{2},g_{3},\ldots ,g_{N}],g_{i}\in \{0,1\}$$

If $g_{i}=1$, it indicates that the *i*th node is selected as the CH; otherwise, it functions as a regular sensor node.

To achieve minimized overall energy expenditure, the fitness function is formulated as presented in Equation (22):

(22) $$\begin{array}{l} F=E_{total}=\sum_{i\in Nodes}E_{i\to CH}+\sum_{j\in CH}E_{j\to \mathrm{Sin}k}\\ E_{i\to CH}=kE_{elec}+k\varepsilon_{fs}d_{i,CH}^{2}\\ E_{CH\to \mathrm{Sin}k}=kE_{elec}+k\varepsilon_{mp}d_{CH,S}^{4} \end{array}$$

where $E_{i\to CH}$ represents the energy consumption from node *i* to its CH CH(*i*), and $E_{CH\to \mathrm{Sin}k}$ represents the energy consumption from CH *j* to the sink.

Randomly select the crossover point c∈{1,2,…,N−1} for single-point crossover. By choosing a crossover point on the parent chromosome and exchanging the tail segments, the continuous gene fragments that perform well are combined into the offspring, with the expectation of forming a more optimal gene combination, thereby accelerating the global search and convergence.

For each $g_{i}\in \{0,1\}$, it is independently executed with a probability of $P_{m}$ to maintain population diversity and prevent premature convergence. Here, $P_{m}=1/N$.

**Figure 6:**
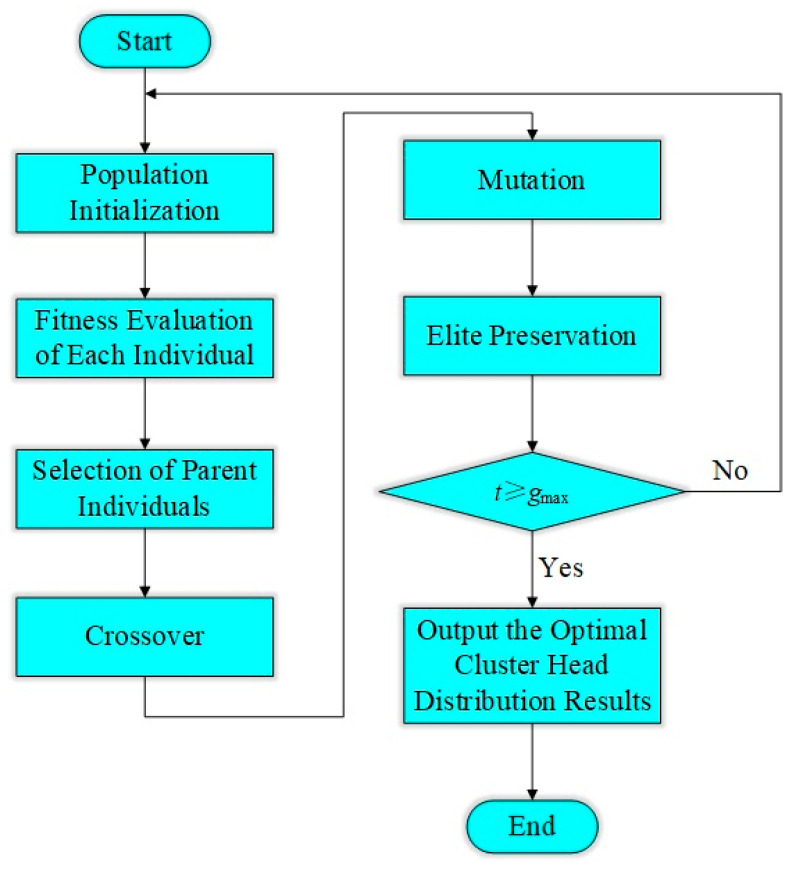
Flowchart of CH selection optimized by GA.

The procedure for optimizing CH selection using the GA is illustrated in Figure 6.

Step 1: Initialize the population. Randomly generate *M* individuals, each of which is a 0/1 binary string for CH selection.

Step 2: Decode and calculate energy consumption, including the communication energy required for data transmission from all member nodes to their respective CH, as well as from the CH to the sink node.

Step 3: Fitness evaluation. Use energy consumption as the fitness criterion, with a smaller value indicating better performance.

Step 4: Select operations. Use the roulette wheel method or tournament selection to choose individuals with lower fitness.

Step 5: Crossover operation. Perform single-point crossover to generate new individuals.

Step 6: Mutation operation. Flip the gene bit 0/1 with probability $P_{m}$.

Step 7: Elite retention. Save the best individuals of this round to the next generation.

Step 8: Determine the stopping condition. The algorithm terminates and outputs the optimal CH configuration when either the maximum number of iterations is reached or the fitness value has converged.

Step 9: Result application. Use the CHs to update the communication structure of the LWSN.

### 4.3. Enhanced TDMA Communication Scheme

Upon completion of the CH selection, the network transitions into the data communication phase. In conventional intra-cluster communication schemes, sensor nodes typically transmit their sensed data to the CH using a static TDMA schedule—i.e., each node is assigned a fixed-length transmission slot—without accounting for real-time data volume, residual energy, or task urgency [51]. Such traditional TDMA-based scheduling strategies often overlook the dynamic heterogeneity in node workload and energy conditions, resulting in suboptimal communication resource utilization and potential energy imbalance across the network.

To improve energy efficiency, enhance the real-time performance of intra-cluster data transmission, and promote equitable task scheduling, this paper introduces a dynamic TDMA scheduling mechanism that leverages task status characteristics observed during the practical operation of the ARTFMR system. This mechanism perceives the task priority and status of nodes, comprehensively considers the current remaining task volume and remaining energy status of nodes, introduces a task-driven node-scoring function, and sorts the transmission sequence of nodes by priority to achieve on-demand allocation of time slot resources.

In contrast to the conventional LEACH protocol, the scheduling mechanism proposed in this study incorporates a task-aware scheduling optimization strategy during the steady-state phase of intra-cluster communication. Traditionally, the LEACH protocol operates in three primary phases within each round: CH selection, cluster formation, and stable data transmission. Moreover, the intra-cluster communication stage adopts a fixed-sequence TDMA scheduling. In contrast, the improved strategy designed in this paper maintains the hierarchical architecture unchanged and optimizes the transmission sequence in the working stage through a node task status awareness mechanism, enhancing the response priority of high-load nodes and slowing down the energy depletion rate of low-energy nodes, thereby achieving dynamic and intelligent resource allocation within the cluster.

Specifically, the comprehensive task-awareness score of the *i*th node is defined as $Q_{i}$, as shown in Equation (23):

(23) $$Q_{i}=\lambda_{Q}\cdot (1-\frac{t_{i,remain}}{t_{\max}})+(1-\lambda_{Q})\cdot (\frac{E_{i}}{E_{avg}})$$

where $t_{i,remain}$ represents the remaining data volume to be sent by node *i* at the current time; $t_{\max}$ represents the maximum remaining task volume among the nodes within the current cluster, which is used for normalization processing; and $\lambda_{Q}$ represents the weighting factor, $\lambda_{Q}\in [0,1]$, used to adjust the relative importance of task urgency and energy balance in the scoring. When $\lambda_{Q}$ is larger, real-time task performance is given more priority; when $\lambda_{Q}$ is smaller, energy balance is emphasized more.

In each round of the data transmission stage, the CH node calculates the $Q_{i}$ of all member nodes according to the above scoring formula and sorts them in descending order of the score. Nodes with higher scores are allocated earlier TDMA time slots, thereby prioritizing the transmission of data from nodes with heavier workloads and greater residual energy. This approach effectively addresses the real-time requirements of data delivery while promoting balanced energy consumption across the network.

By introducing task-aware scheduling modeling, not only is the difference in energy depletion between nodes effectively reduced, the intra-cluster communication delay caused by load congestion is also alleviated, providing strong support for extending the network’s life cycle and enhancing data transmission efficiency.

## 5. Simulation Experiment

### 5.1. Simulation Environment Setup

To assess the effectiveness of the proposed energy-balanced non-uniform node deployment scheme and GAECRPQ protocol, a series of simulation experiments were carried out in MATLAB R2022b, with performance comparisons made against six baseline protocols: energy-aware distributed unequal clustering (EADUC) [52], energy-aware unequal clustering algorithm (EAUCA) [53], energy-balanced unequal clustering (EBUC) [54], energy-efficient unequal clustering (EEUC) [55], LEACH [49], and LEACH-C [56].

The corresponding simulation parameters are summarized in Table 1. The initial energy of each sensor node is designated as 0.5 J. It is further assumed that all nodes are provided with sufficient time to complete data transmission during each operational cycle. Throughout the simulation process, only the energy consumed during wireless communication is taken into account, whereas the energy expenditure associated with sensing and data processing operations is disregarded. A full operational cycle is defined as the complete sequence wherein a sensor node acquires data and transmits it to the sink node via the CH. The network lifetime is measured as the total number of operational cycles completed before all sensor nodes deplete their energy reserves and become non-functional.

To provide a comprehensive assessment of the performance advantages of the GAECRPQ algorithm, this study employs the following nine metrics as evaluation criteria for the routing protocol: network coverage, CH energy consumption ratio, the variation in surviving node count across operational rounds, the round at which the first node failure occurs (FDN), the round at which half of the nodes have failed (HDN), the round at which all nodes have failed (ADN), total energy consumption over the course of operations, the cumulative number of data packets received by the base station over time, and average transmission delay under different node scales.

### 5.2. Analysis of Simulation Experiment Results

**Figure 7:**
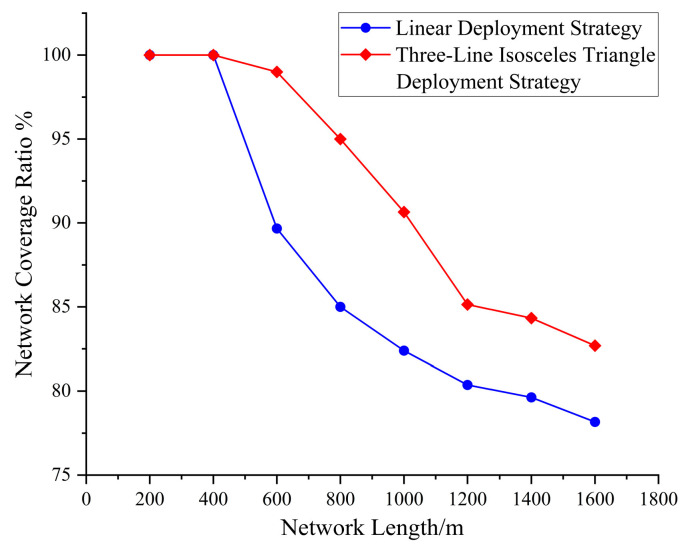
Network coverage rate.

Figure 7 illustrates a comparative evaluation of the network coverage performance between the proposed three-line isosceles triangle deployment strategy and the conventional linear deployment method. Given an equal total number of sensor nodes, the proposed approach markedly enhances the overall coverage of the network. Furthermore, to achieve the same level of coverage, it requires fewer sensor nodes, demonstrating superior efficiency in node utilization and coverage effectiveness. These results substantiate the comprehensive advantages of the three-line isosceles triangle deployment method in optimizing resource allocation and enhancing network performance.

**Figure 8:**
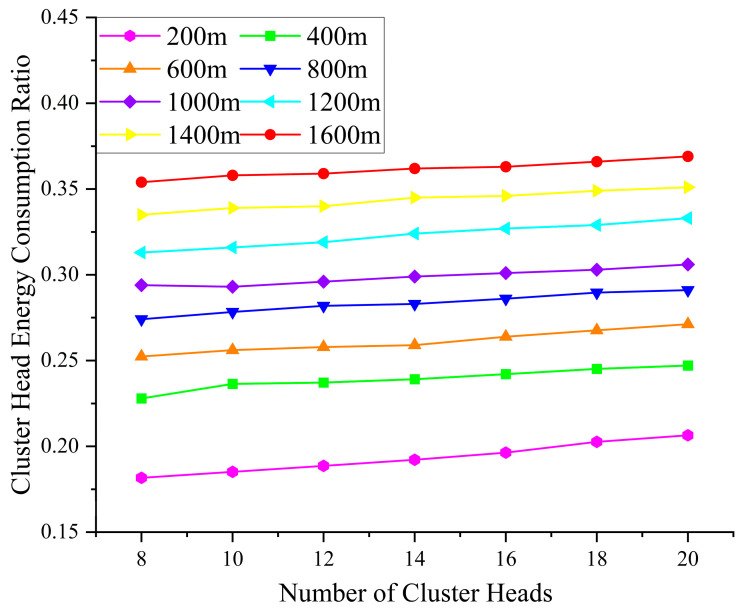
Energy consumption ratio of CHs.

Figure 8 illustrates the relationship between the number of clusters and the energy consumption ratio of CHs under varying network lengths, based on 2000 simulation rounds using the energy consumption-balanced non-uniform deployment strategy. As shown in the figure, under the condition of a fixed number of clusters, the energy consumption ratio of CHs increases with the network length. This trend is primarily attributed to the increased communication distance among sensor nodes, which leads to higher energy consumption during data transmission.

Furthermore, as the network length increases incrementally from 200 m to 1600 m, the optimal number of clusters corresponding to the lowest CH energy consumption is observed to be 8, 8, 8, 8, 10, 8, 8, and 8, respectively. This non-linear trend suggests that the optimal cluster count is not exclusively dictated by the network length but is also affected by various parameters, such as transmission range, topological structure, and intra-cluster data aggregation burden. Therefore, in real-world deployment scenarios, the cluster count should be adaptively tuned according to the network length and node distribution patterns to reduce CH energy expenditure and further optimize the overall energy efficiency of the WSN.

In order to comprehensively assess the effectiveness of the proposed GAECRPQ protocol, a performance comparison was conducted against a set of representative benchmark protocols: EADUC, EAUCA, EBUC, EEUC, LEACH, and LEACH-C.

**Figure 9:**
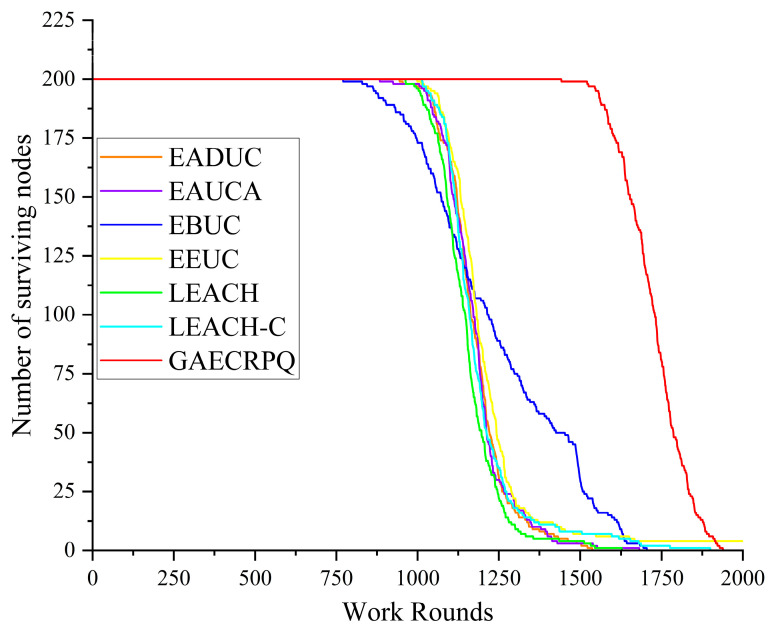
Comparison of the number of surviving nodes in the network and working rounds.

The number of surviving nodes is a key indicator for evaluating the performance of routing protocols, as it reflects the network’s operational lifetime. A slower decline in the number of surviving nodes indicates a longer network lifespan and greater system endurance. Assuming that all sensor nodes start with the same initial energy, the survival performance of seven different protocols was simulated. The results are presented in Figure 9.

As shown in the simulation results in Figure 9, with a total of 200 sensor nodes, the EBUC protocol begins to exhibit node energy depletion around the 800th round. All nodes in the six protocols—EADUC, EAUCA, EBUC, EEUC, LEACH, and LEACH-C—completely deplete their energy within 1750 rounds, reflecting relatively short network lifetimes. In contrast, the GAECRPQ protocol proposed in this study demonstrates significantly better performance, with all nodes maintaining functionality until approximately the 1880th round. This marked extension of network lifetime provides compelling evidence of the proposed protocol’s effectiveness in achieving balanced energy consumption and improving overall system stability.

As summarized in Table 2, the proposed GAECRPQ protocol simultaneously achieves the best performance in all three evaluation metrics—FDN, HDN, and ADN—thereby substantiating its effectiveness in prolonging network lifetime, enhancing system stability, and achieving balanced energy consumption. Concretely, compared with the six benchmark protocols, GAECRPQ defers the round at which the first node dies (FDN) by 53%, 62%, 86%, 45%, 50%, and 42%, respectively. Correspondingly, the effective network lifetime, measured by the half-node-death (HDN) round, is extended by 49%, 47%, 42%, 46%, 51%, and 49%. These empirical results provide compelling evidence that GAECRPQ substantially outperforms existing schemes in sustaining long-term operational efficiency and stability.

**Figure 10:**
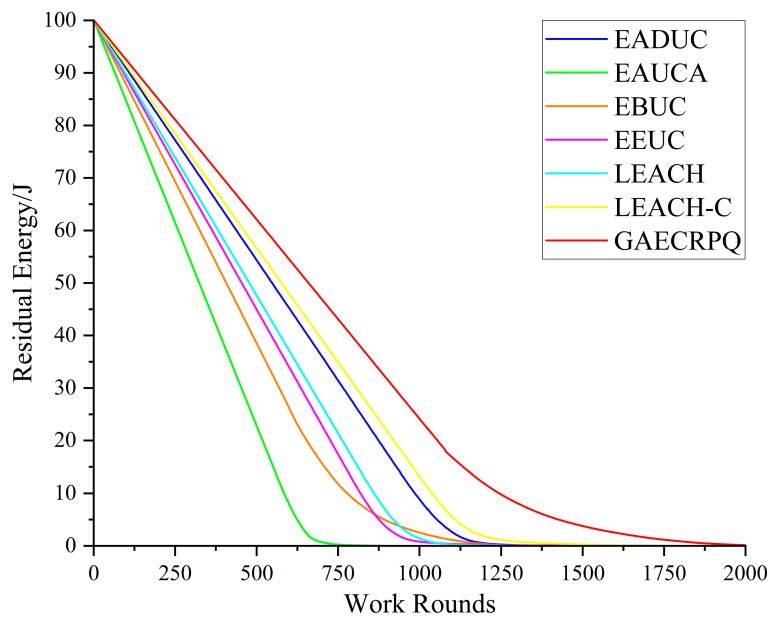
Comparison of total energy consumption of network nodes and working rounds.

Although the number of surviving nodes offers a general indication of network status, it does not fully capture the dynamics of overall energy consumption. Therefore, it is essential to analyze variations in the total residual energy to provide a more comprehensive assessment of each protocol’s energy efficiency during both data transmission and control signaling operations. Figure 10 illustrates a comparative analysis of the residual energy levels of network nodes across seven distinct protocols over a range of operational rounds. The simulation results reveal that the GAECRPQ protocol exhibits the slowest rate of energy decline throughout the operational process, indicating the lowest overall energy consumption rate. Under the condition of an identical total number of nodes, the EADUC, EAUCA, EBUC, EEUC, LEACH, and LEACH-C protocols deplete all node energy by approximately the 1250th round, thereby terminating network operation. In contrast, the GAECRPQ protocol retains residual energy in some nodes beyond this point, resulting in an extended operational period and a network lifetime approximately 40% longer than those of the other protocols. The results clearly indicate that the GAECRPQ protocol surpasses the six benchmark protocols in terms of energy utilization efficiency, demonstrating superior energy management capabilities and strong potential for extending the overall network lifetime.

**Figure 11:**
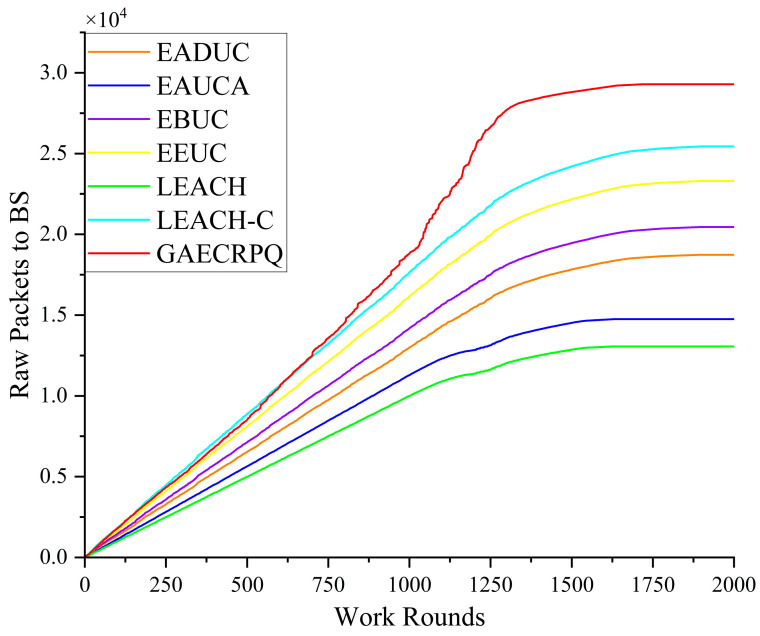
Comparison of data packets received by the base station and working rounds.

Figure 11 shows the trend of network throughput under seven protocols as the network operation time changes. As illustrated in Figure 11, the network throughput of all evaluated protocols progressively increases with the number of operational rounds and reaches its respective peak near the end of the network’s lifetime. This trend indicates that each protocol is capable of maintaining satisfactory throughput performance during the network’s stable operational phase. Meanwhile, the GAECRPQ protocol maintains a consistently high level of network throughput throughout the entire operation process, significantly outperforming the other six protocols. Notably, as the scale of network nodes increases, the throughput advantage of the GAECRPQ protocol becomes increasingly pronounced, highlighting its superior scalability and transmission efficiency in large-scale wireless sensor network deployments.

Since intra-cluster communication adopts the TDMA mechanism, during each round of the data transmission phase, the CH node calculates the $Q_{i}$ value of all member nodes based on the aforementioned scoring formula and ranks them in descending order. This approach ensures that nodes with a higher load and residual energy are prioritized for transmission, thereby simultaneously addressing the real-time requirements of data transmission and achieving a more balanced energy consumption among nodes. Accordingly, a series of simulation experiments were conducted to assess the delay performance. The number of deployed nodes under each of the seven protocols was progressively increased from 50 to 250 in steps of 20, with the duration of each time slot fixed at 2 ms. Each protocol was executed over 200 independent simulation runs, and the average outcomes were recorded for performance comparison.

**Figure 12:**
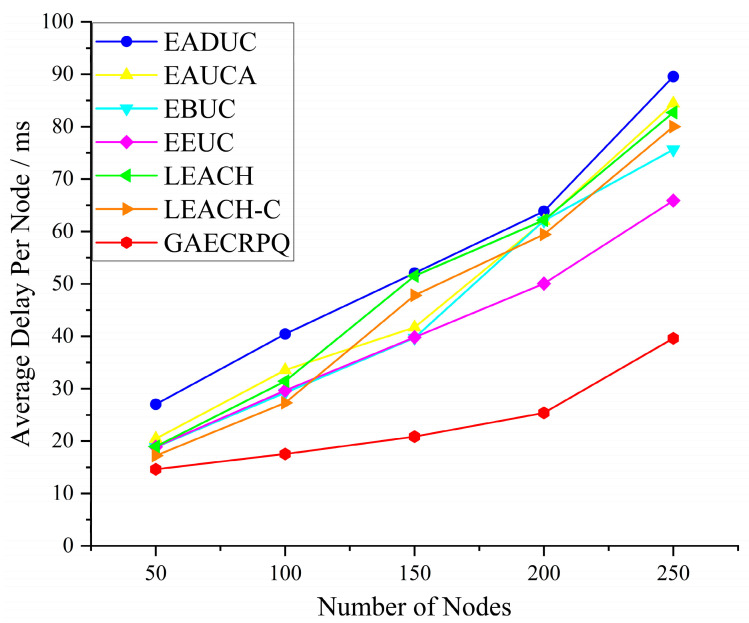
Average delay with different node numbers.

Figure 12 illustrates the relationship between the number of deployed nodes and the average end-to-end delay per node across seven different routing protocols. The simulation outcomes reveal a consistent upward trend in average delay as the network scale increases. Nevertheless, the rate of delay escalation differs markedly among the evaluated protocols. Specifically, the EADUC, EAUCA, EBUC, EEUC, LEACH, and LEACH-C protocols exhibit a rapid increase in average delay, whereas the GAECRPQ protocol demonstrates a much slower growth trend. When the number of network nodes reaches 250, the GAECRPQ protocol achieves reductions in average node delay of 55.77%, 53.07%, 47.61%, 39.87%, 52.08%, and 50.48% compared to the ADUC, EAUCA, EBUC, EEUC, LEACH, and LEACH-C protocols, respectively. These results indicate that the GAECRPQ protocol exhibits superior scalability and delay performance, particularly in large-scale WSN deployments.

The simulation outcomes evaluate the proposed deployment strategy from multiple perspectives, including network lifetime, total energy consumption, and residual energy distribution. Experimental analysis demonstrates that the proposed deployment model significantly enhances energy utilization efficiency, prolongs the network lifetime, and improves the overall network stability.

## 6. Conclusions

This paper proposes the GAECRPQ routing protocol, specifically tailored for LWSNs within the Automated Railway Track Fastener Maintenance Robot (ARTFMR) system under railway environments. During the clustering phase, both residual energy and transmission distance are jointly evaluated for CH selection. In each round of CH selection, an adaptive weighting scheme is applied to combine the average residual energy of nodes with their relative distance to the sink node. The CH selection probability is subsequently optimized via a GA, achieving dynamic adaptation and equitable CH role distribution. Additionally, by incorporating real-time task information from ARTFMR operations, the protocol introduces a task-aware TDMA scheduling scheme to prioritize communication based on task urgency. This enhances real-time responsiveness and energy utilization efficiency while adhering to latency constraints. Simulation results validate that GAECRPQ outperforms six baseline routing protocols in terms of network longevity and energy performance.

In subsequent research, we will further refine the collaborative control mechanisms of the ARTFMR system to better align with practical operational requirements. First, within the context of mobile aggregation nodes, we plan to investigate a variety of mobility strategies and scheduling frameworks aimed at mitigating energy consumption in high-traffic areas and improving the real-time efficiency of data collection. Second, in large-scale network environments involving multi-robot collaboration, we will focus on enhancing scalability and reducing latency under conditions of high-density node deployment and concurrent multi-task execution. This will ensure the protocol’s adaptability, robustness, and broad applicability in complex railway maintenance scenarios. Finally, we intend to explore cross-layer optimization combined with task-driven integration. By synthesizing information across the routing layer, MAC layer, and task layer, we aim to achieve a coordinated optimization of energy efficiency, latency control, and task prioritization, thereby delivering more intelligent and adaptive communication support for the ARTFMR system.

## Figures and Tables

**Table 1:** Network simulation parameter settings.

Parameter Symbol	Parameter Description	Value Assignment
$E_{0}$	Initial Energy	0.5 J
$E_{elec}$	Unit energy Consumption	50 nJ $\cdot {\mathrm{bit}}^{-1}$
$\varepsilon_{fs}$	Energy Consumption Coefficient	10 pJ $\cdot {(\mathrm{bit}\cdot m^{2})}^{-1}$
$\varepsilon_{mp}$	Energy Consumption Coefficient	0.0013 pJ $\cdot {(\mathrm{bit}\cdot m^{4})}^{-1}$
*k*	Packet Length	1024 bit
*N*	Total Number of Nodes	240
$d_{0}$	Communication Distance Threshold	87.7 m
*p*	Expected Cluster Head Proportion	0.05

**Table 2:** Comparison of FDN, HDN, and ADN.

Protocol	FDN	HDN	ADN
EADUC	945	1163	1543
EAUCA	890	1174	1687
EBUC	776	1215	1708
EEUC	999	1182	1681
LEACH	963	1147	1630
LEACH-C	1015	1160	1905
GAECRPQ	1445	1729	1939

## Data Availability

Data are contained within the article.
